# Recent Advances in Functional Polyisoprene: From Precision Synthesis to Smart Elastomers

**DOI:** 10.3390/polym18202474

**Published:** 2026-10-10

**Authors:** Ji Ma, Guangye Liu, Dandan Liu, Naixiu Ding, Lixia He

**Affiliations:** 1 College of Polymer Science and Engineering, Qingdao University of Science and Technology, Qingdao 266042, China; 02630@qust.edu.cn; 2 Engineering Research Center of High-Performance Polymer and Molding Technology, Ministry of Education, Qingdao University of Science and Technology, Qingdao 266042, China; 00515@qust.edu.cn (G.L.); 02095@qust.edu.cn (N.D.)

**Keywords:** polyisoprene, precision polymerization, polymer functionalization, smart elastomers

## Abstract

Polyisoprene is an important elastomer with a tunable microstructure and a hydrocarbon backbone analogous to the rubber component of natural rubber. However, conventional synthetic polyisoprene lacks the complex intermolecular interactions associated with non-rubber components in natural rubber, limiting its mechanical performance and functional versatility. Recent advances in precision polymerization and functionalization provide new opportunities for developing high-performance and adaptive polyisoprene elastomers. This review summarizes progress in polyisoprene precision synthesis, microstructure control, and functionalization, with emphasis on the relationships among molecular structure, chemical reactivity, network architecture, and macroscopic properties. Coordination and living/controlled polymerization enable regulation of stereoregularity, molecular weight, and chain-end functionality, while epoxidation, hydroxyl functionalization, halogenation, click chemistry, and grafting provide versatile routes to functional polyisoprene. Dynamic covalent bonds and supramolecular interactions further enable reinforced, self-healing, reprocessable, recyclable, and stimuli-responsive elastomers. Importantly, their overall performance depends on balancing chain mobility, intermolecular interactions, network stability, and bond-exchange dynamics. Finally, emerging applications and challenges in multifunctionality and sustainability are discussed, providing molecular-design principles for next-generation functional polyisoprene elastomers.

## 1. Introduction

Natural rubber (NR) is one of the most important renewable elastomers and has been widely used in tires, gloves, waterproof materials, and medical devices due to its excellent elasticity, resilience, tensile strength, and strain-induced crystallization behavior [1,2]. However, the limited geographical distribution of rubber cultivation, long growth cycles, and vulnerability to environmental changes restrict the stable supply of NR. Therefore, synthetic elastomers capable of reproducing or surpassing the properties of NR have attracted increasing attention. Polyisoprene (PIP), consisting of repeating isoprene units, is considered one of the most promising alternatives because of its identical chemical backbone and tunable microstructures. Depending on stereoregularity and polymerization pathways, PIP exhibits cis-1,4-, trans-1,4-, 1,2-, and 3,4-configurations, which strongly influence chain mobility, crystallization behavior, and mechanical performance [3,4]. Among them, cis-1,4-polyisoprene (cis-1,4-PIP) closely resembles NR and exhibits excellent elasticity and durability. Recent advances in transition-metal catalysis, rare-earth catalytic systems, and precision polymerization have enabled the synthesis of highly stereoregular cis-1,4-PIP with controlled molecular characteristics, providing new opportunities for advanced elastomer design [5,6]. Moreover, the chemical versatility of PIP allows the incorporation of functional groups and dynamic interactions, facilitating the development of adaptive and stimuli-responsive elastomers [7].

Although synthetic cis-1,4-PIP possesses a similar backbone structure to NR, its mechanical durability is generally inferior due to the absence of NR-specific non-rubber components. Proteins, phospholipids, and fatty acids located at NR chain ends and interfaces form complex physical networks through hydrogen bonding, ionic interactions, and supramolecular associations, contributing to enhanced strain-induced crystallization, reinforcement, and fatigue resistance [8,9,10,11]. Therefore, introducing functional moieties into PIP chains has become an effective strategy to mimic the reinforcing effects of NR components while providing additional functionalities [12].

Recent progress in molecular engineering has greatly expanded the functionalization strategies of PIP, including copolymerization, post-polymerization modification, and dynamic network construction. For example, Cui et al. introduced methoxy and pyridine functionalities into cis-1,4-PIP through copolymerization with polar diene derivatives, improving polarity and hydrophilicity [13,14,15]. Gong et al. reported hydroxyl-functionalized PIP through copolymerization with hydroxyl-containing myrcene derivatives, where hydroxyl groups enhanced filler–polymer interactions and improved mechanical performance [16]. These studies demonstrate that rational molecular design provides an effective approach for overcoming the intrinsic limitations of conventional PIP. Several previous reviews have summarized the development of synthetic polyisoprene from different perspectives. In particular, Cruz-Morales et al. reviewed synthetic polyisoprene rubber as a mimic of natural rubber, with emphasis on its synthesis, structural characteristics, nanocomposites, and applications [17].

**Figure 1:**
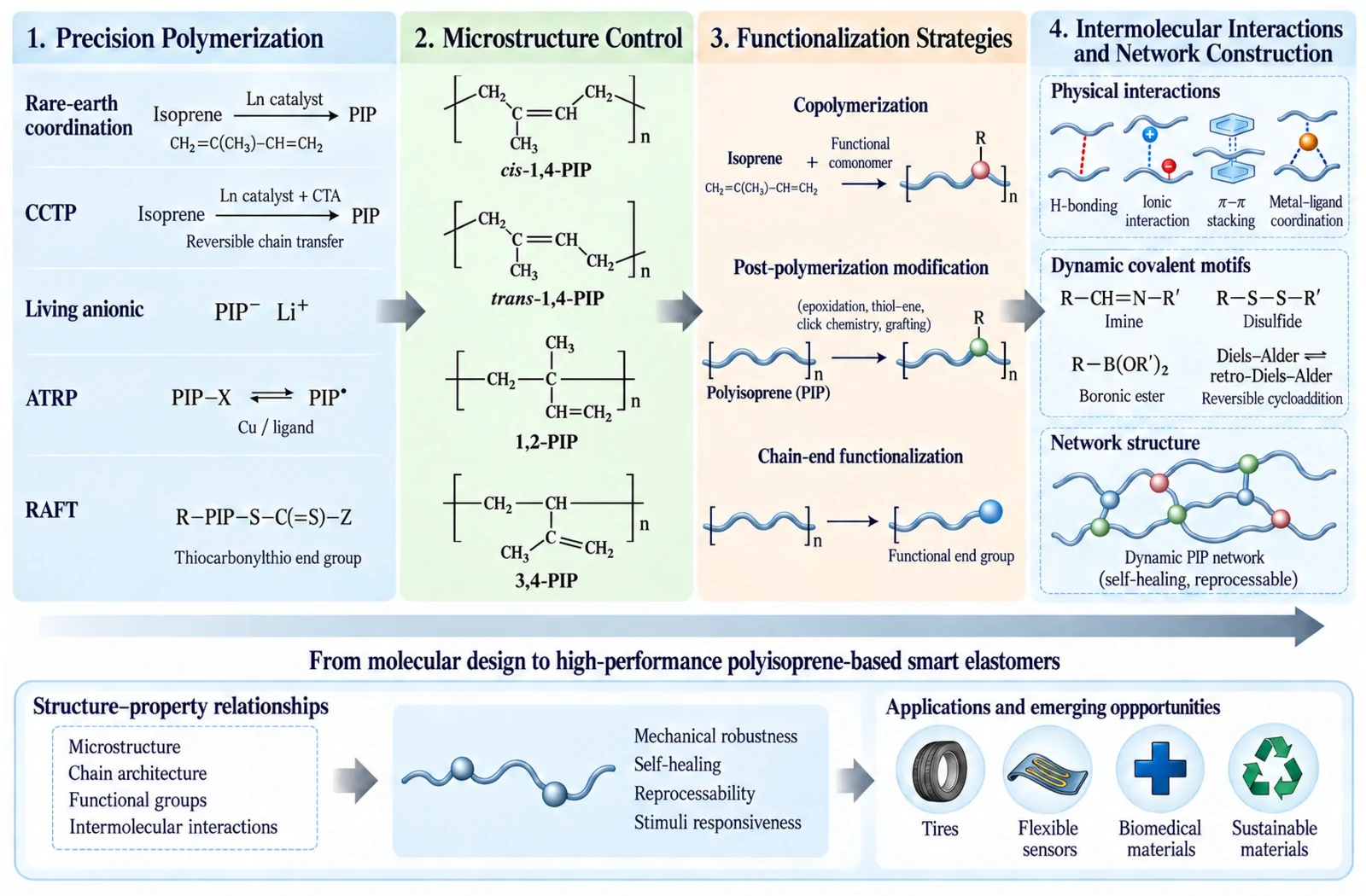
Hierarchical molecular-design framework for functional polyisoprene linking precision synthesis and microstructure control to functionalization, intermolecular interactions, network architecture, macroscopic properties, and smart-elastomer functions.

Accordingly, the objective of this review is to establish an integrated molecular-design perspective for functional polyisoprene by linking precision synthesis and microstructure control with chemical functionalization, intermolecular interactions, network architecture, and macroscopic functional properties. Recent advances in precision polymerization have enabled precise control over molecular weight, dispersity, stereoregularity, sequence structure, and chain-end functionality [18,19,20,21]. Importantly, polymer synthesis, functionalization, and network construction should not be regarded as independent processes, because polymerization determines PIP microstructure and reactive sites, functionalization regulates intermolecular interactions, and network architecture ultimately governs macroscopic properties. As depicted in Figure 1, the development of functional PIP can be viewed as a hierarchical molecular-design process, progressing from precision polymerization and microstructure control to chemical functionalization, intermolecular interactions, network construction and ultimately macroscopic properties and functional performance.

Rather than treating polymerization, chemical modification, and functional elastomers as isolated topics, this review integrates these processes within a unified structure–property framework. Particular emphasis is placed on how polymerization-controlled microstructure determines the availability and accessibility of reactive sites, how subsequent functionalization regulates intermolecular interactions, and how these interactions are translated through network architecture into mechanical, dynamic, and stimuli-responsive properties. This framework enables different synthetic and functionalization strategies to be compared in terms of their molecular consequences and performance trade-offs, thereby providing molecular-design principles for the development of next-generation functional PIP elastomers.

## 2. Precision Synthesis and Microstructure Control of Polyisoprene

### 2.1. Coordination Polymerization

Coordination polymerization is a key strategy for the stereoselective polymerization of conjugated dienes, enabling the synthesis of polyisoprene with well-defined microstructures and properties approaching those of natural rubber. Among the available polymerization techniques, coordination polymerization is particularly attractive because it can precisely regulate the stereochemistry of the polymer backbone, with the cis-1,4 content typically exceeding 97%, thereby imparting excellent elasticity, resilience, and mechanical performance comparable to those of natural rubber [6,22]. Since the pioneering studies on Ziegler–Natta catalysts in the 1950s, tremendous progress has been achieved in catalyst design, evolving from conventional titanium-based systems to highly efficient rare-earth and transition-metal catalysts capable of producing polyisoprene with excellent stereoregularity and narrow molecular-weight distributions [23].

Rare-earth catalytic systems have attracted significant attention due to their high activity and excellent stereoselectivity [24,25]. Compared with conventional Ziegler–Natta catalysts, rare-earth catalysts provide a unique opportunity to directly introduce functional groups into polyisoprene while maintaining a high cis-1,4 content, thereby overcoming one of the major limitations of traditional coordination polymerization. One of the most significant advances in recent years has been the direct copolymerization of isoprene with polar diene derivatives. Mecking and co-workers demonstrated that industrially relevant Nd-based catalytic systems are capable of copolymerizing isoprene with nitrogen- and sulfur-containing diene monomers, producing functionalized polyisoprene with high cis-1,4 regularity while incorporating polar functionalities into the polymer backbone [26]. Subsequently, the same group employed a borohydride neodymium catalyst in combination with functional dialkylmagnesium chain-transfer reagents to prepare polyisoprene containing functional groups both along the polymer backbone and at the chain termini, providing a versatile strategy for the synthesis of multifunctional elastomers through coordination polymerization.

Considerable efforts have also been devoted to expanding the monomer scope of rare-earth-catalyzed polymerization. Cui and co-workers achieved highly stereoselective copolymerization of isoprene with a pyridine-functionalized diene using an yttrium-based catalytic system, enabling tunable polar-monomer incorporation while maintaining >97% cis-1,4 selectivity [14]. Subsequently, Cui et al. reported a facile strategy to prepare star polyisoprene using an isocyanate cross-linker to terminate the cis-1,4-selective living coordination polymerization of isoprene mediated by an yttrium-based catalyst. The obtained star polymers possessed narrow molecular-weight distributions and high coupling efficiencies, illustrating the capability of coordination polymerization to precisely control polymer topology in addition to stereochemistry [27].

Despite these advances, direct copolymerization of highly polar monomers remains challenging because oxygen- or nitrogen-containing functional groups can strongly coordinate to rare-earth metal centers and deactivate the catalyst. To address this issue, Xu and co-workers developed a practical protection strategy in which ester-, ether-, hydroxyl-, and imide-containing monomers were temporarily masked by alkylaluminum reagents prior to polymerization. This approach effectively suppressed catalyst poisoning while enabling the successful copolymerization of a broad range of polar monomers with isoprene, greatly expanding the scope of functional monomers compatible with industrial rare-earth catalytic systems [28,29,30,31].

Beyond backbone functionalization, coordination polymerization has recently been integrated with coordinative chain transfer polymerization (CCTP), providing an efficient route toward end-functionalized polyisoprene. In CCTP systems, reversible chain transfer between the active catalyst and organometallic chain-transfer agents allows multiple polymer chains to be generated from a single catalytic center while maintaining excellent control over molecular weight and stereoregularity [32,33,34]. By combining functional chain-transfer agents with living coordination polymerization, polyisoprene bearing well-defined functional chain ends or dual-functionalized chain termini can be readily prepared with high functionalization efficiency and excellent scalability, making this strategy particularly attractive for industrial production of functional elastomers and advanced polymer architectures [35]. More recently, Zhang et al. developed a neodymium-mediated CCTP strategy for the synthesis of α, ω-end hetero-functionalized polyisoprene. α-End functionality was introduced using copolymerizable heteroatom-containing diene derivatives during polymerization, while ω-end functionalization was subsequently achieved through either functional diene incorporation or reactions of the reactive allyl–metal chain ends with small molecules. This approach demonstrates the potential of CCTP for constructing PIP with independently tunable functionalities at both chain termini [36]. A recent review by Liu et al. further summarized advances in end-functionalization during CCTP of conjugated dienes, highlighting the use of highly reactive metal–carbon bonds generated during chain transfer as a versatile platform for in situ chain-end functionalization [37].

### 2.2. Living and Controlled Polymerization

Living and controlled polymerization provides a versatile platform for the precision synthesis of functional polyisoprene by enabling control over molecular weight, dispersity, macromolecular architecture, and chain-end functionality [38,39]. Among the various living polymerization techniques, anionic polymerization remains one of the most effective approaches for the synthesis of structurally well-defined polyisoprene. Because chain termination and irreversible chain-transfer reactions are essentially absent under rigorously controlled conditions, living anionic polymerization affords polyisoprene with predictable molecular weights, narrow dispersities, and excellent chain-end fidelity [40].

More importantly, the living polyisoprenyl chain ends can be efficiently functionalized with various electrophiles, allowing the introduction of hydroxyl, carboxyl, amino, epoxy, and other functional groups. This capability has enabled the preparation of telechelic, star-shaped, grafted, and other architecturally complex PIP derivatives that serve as versatile precursors for subsequent functionalization and network construction [41,42]. Gates and co-workers further developed phosphorus-containing functional isoprene monomers through anionic polymerization, introducing coordination-active phosphorus groups into polyisoprene and providing new opportunities for biomimetic cross-linking and metal–polymer hybrid materials [43].

Despite its high structural precision, living anionic polymerization requires stringent reaction conditions and exhibits limited tolerance toward polar functional groups. Controlled radical polymerization (CRP), particularly atom transfer radical polymerization (ATRP) and reversible addition–fragmentation chain transfer (RAFT) polymerization, provides a complementary approach with broader functional-group tolerance and milder reaction conditions [44,45]. ATRP has been demonstrated for controlled isoprene polymerization [45], whereas RAFT has been more widely employed to construct functional PIP-containing block and multiblock copolymers and to introduce reactive chain ends for subsequent modification [46,47]. Living anionic polymerization offers superior control over molecular characteristics and chain-end functionality but is constrained by stringent reaction conditions and limited compatibility with polar functionalities. In contrast, controlled radical methods provide greater chemical versatility and functional-group tolerance, although their control over PIP microstructure and stereoregularity is generally more limited. These complementary characteristics make the selection of polymerization strategy an important determinant of both structural precision and subsequent functionalization pathways. The major polymerization strategies for functional PIP are compared in Table 1 in terms of structural control, functionalization mode, advantages, limitations, and representative PIP-related applications.

### 2.3. Microstructure Control and Chemical Reactivity

The microstructure of polyisoprene, including the relative distribution of cis-1,4-, trans-1,4-, 1,2-, and 3,4-units, plays a decisive role in determining not only the physical properties of the polymer but also its chemical reactivity and subsequent functionalization behavior. Advances in stereoselective polymerization have enabled precise regulation of these structural units, providing an effective strategy for tailoring the density and accessibility of reactive sites while simultaneously optimizing the mechanical performance of the resulting elastomers [52,53]. Therefore, microstructure engineering has become an indispensable bridge connecting precision polymerization with functional material design.

Among the different microstructures, cis-1,4-polyisoprene possesses a highly flexible backbone and a low glass-transition temperature, making it primarily responsible for the outstanding elasticity and resilience of rubber materials. The highly regular cis configuration also facilitates strain-induced crystallization, thereby significantly improving tensile strength and fatigue resistance [2,54]. However, because the carbon–carbon double bonds are embedded within a relatively flexible hydrocarbon backbone and the polymer exhibits intrinsically low polarity, the accessibility of reactive sites toward certain chemical transformations is relatively limited. Consequently, post-polymerization functionalization of high cis-1,4-polyisoprene generally relies on selective reactions of the residual carbon–carbon double bonds, including epoxidation, thiol–ene addition, hydrogenation, halogenation, and grafting reactions [51,55].

In contrast, 3,4-polyisoprene contains pendant vinyl groups that are considerably more accessible than the internal double bonds in cis-1,4-polyisoprene. These pendant unsaturated groups serve as highly reactive sites for a wide variety of chemical transformations, including thiol–ene click reactions, radical additions, epoxidation, hydrosilylation, and photo-induced cross-linking [53,56,57]. As a result, increasing the 3,4-unit content can substantially enhance the functionalization efficiency and broaden the range of accessible chemical modifications. In recent years, catalyst-controlled synthesis of high-3,4-polyisoprene has therefore attracted increasing interest as a promising strategy for constructing multifunctional elastomers with improved processability and higher densities of reactive functional groups.

Compared with the highly elastic cis-1,4 structure, trans-1,4-polyisoprene exhibits a more ordered chain conformation and significantly higher crystallinity. This unique microstructure endows the material with superior dimensional stability, stiffness, and mechanical strength, while also affecting solvent diffusion, reaction kinetics, and cross-linking behavior during chemical modification [58,59]. Consequently, trans-rich polyisoprene has found increasing applications in shape-memory materials, high-strength elastomers, and semicrystalline functional polymers, where the balance between crystallinity and chemical reactivity plays a critical role.

**Figure 2:**
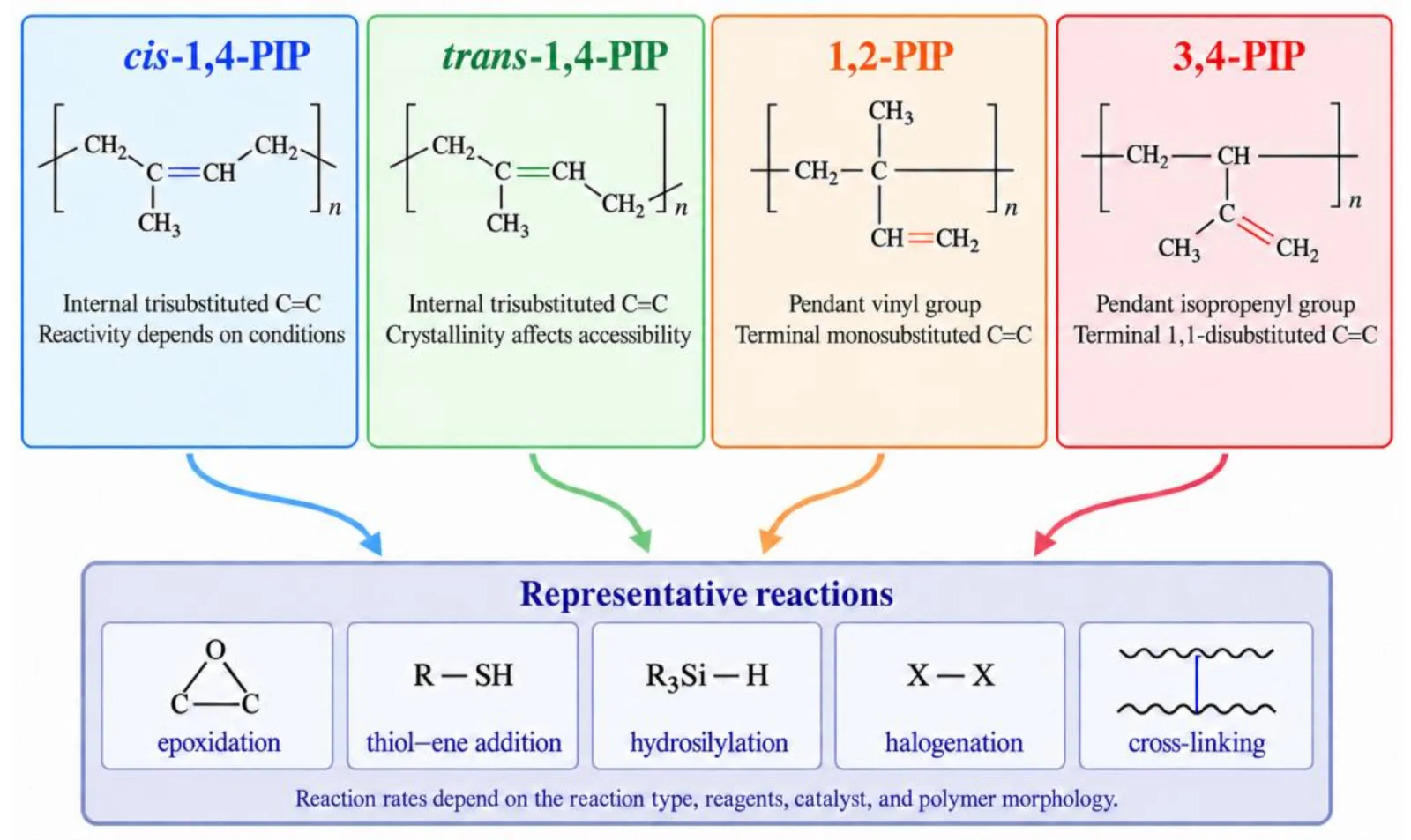
Influence of polyisoprene microstructure on chain characteristics, reactive-site accessibility, and subsequent functionalization pathways.

In addition to stereoregularity, the incorporation of 1,2-units also provides terminal vinyl functionalities that can participate in a variety of post-polymerization reactions. Although the 1,2-content in conventional polyisoprene is generally low, recent catalyst design has enabled precise regulation of 1,2-selectivity, providing additional opportunities for introducing functional groups and constructing cross-linked or branched polymer architectures [52]. As summarized in Figure 2, the microstructure of PIP provides a direct link between polymer synthesis and subsequent functionalization. The cis-1,4 and trans-1,4 configurations primarily determine chain flexibility, crystallization behavior, and mechanical properties, whereas the pendant vinyl groups in 3,4- and 1,2-PIP provide more accessible reactive sites for post-polymerization modification. Therefore, PIP microstructure should be regarded not only as a determinant of physical properties but also as a key molecular parameter governing reactive-site accessibility and subsequent functionalization pathways.

The above polymerization strategies provide distinct advantages in controlling PIP microstructure, molecular weight, chain-end functionality, and processability. Coordination polymerization, particularly using rare-earth catalysts, remains the most effective approach for achieving highly stereoregular cis-1,4-polyisoprene due to its excellent regio- and stereoselectivity. In contrast, living anionic polymerization provides superior control over molecular weight distribution and chain-end fidelity, although its stringent reaction conditions limit large-scale implementation. CCTP combines high catalyst efficiency with effective control over molecular weight and stereoregularity, making it attractive for scalable synthesis. Controlled radical polymerization methods such as ATRP and RAFT exhibit excellent functional-group tolerance and versatility for introducing diverse functional moieties, but their ability to precisely regulate PIP microstructure is relatively limited. Thus, the selection of a polymerization strategy should depend on the targeted balance among stereoregularity, molecular precision, functional compatibility, and process scalability, as summarized in Table 1.

## 3. Functionalization Strategies of Polyisoprene

Functionalization represents a key molecular-engineering strategy for expanding the chemical diversity and application scope of PIP. Although PIP possesses excellent elasticity and mechanical resilience arising from its flexible hydrocarbon backbone, its intrinsically nonpolar nature, low surface energy, and limited chemical reactivity considerably restrict its applications in advanced functional materials. Consequently, introducing functional groups into the polymer backbone or chain ends has become an indispensable approach for tailoring its physicochemical properties and constructing multifunctional elastomeric systems [7,60,61]. Depending on the modification strategy, the polarity, intermolecular interactions, thermal stability, oil resistance, gas barrier properties, adhesion, and compatibility of PIP can be significantly improved while simultaneously providing reactive sites for subsequent molecular engineering.

Functional PIP can be obtained through direct transformation of backbone unsaturation, incorporation of functional comonomers, chain-end modification, click reactions, grafting, and subsequent network construction. Representative strategies include epoxidation, hydroxylation, halogenation, click chemistry, grafting reactions, and dynamic covalent network construction. These methodologies not only enrich the molecular diversity of polyisoprene but also provide versatile synthetic platforms for preparing smart elastomers with self-healing, recyclable, stimuli-responsive, and biomedical functionalities [55,62,63,64].

### 3.1. Epoxidation

Epoxidation is one of the most established post-polymerization strategies for functionalizing polyisoprene. By selectively converting carbon–carbon double bonds into epoxide groups using peracids or peroxide-based systems, the polarity and chemical reactivity of PIP can be substantially increased while largely preserving its polymer backbone [65,66,67,68]. The resulting epoxidized PIP generally exhibits enhanced intermolecular interactions, oil resistance, gas-barrier properties, and compatibility with polar fillers, making epoxidation an effective route for tuning both interfacial and bulk properties.

More importantly, the strained epoxide groups provide reactive sites for subsequent chemical transformations. Nucleophilic ring-opening reactions with amines, alcohols, thiols, and carboxylic acids enable the introduction of hydroxyl, amino, carboxyl, sulfide, and other functionalities. Epoxidized PIP can therefore serve as a versatile intermediate for constructing grafted, branched, cross-linked, and supramolecular polymer architectures. Several representative studies have demonstrated the versatility of epoxidation in advanced polymer architecture design. Gauthier and co-workers prepared highly branched polyisoprene by first introducing randomly distributed epoxy groups onto high cis-1,4-polyisoprene through controlled epoxidation, followed by coupling reactions between the epoxidized polymer and living polyisoprenyl lithium chains. This strategy enabled the efficient synthesis of highly branched polyisoprene with well-controlled molecular architectures while maintaining the desirable elastomeric properties of the parent polymer [69]. Zhang and co-workers further employed epoxidized polyisoprene as a multifunctional macroinitiator for grafting polystyrene side chains through sequential epoxidation and epoxide ring-opening reactions, ultimately constructing cylindrical polymer brushes with dendritic side-chain architectures [70]. These studies highlight that epoxidation serves not merely as a polarity-enhancing modification but also as an effective route toward sophisticated polymer topologies.

Despite these advantages, careful control of the epoxidation degree remains essential. Excessive oxidation may induce chain scission, undesired intermolecular cross-linking, or deterioration of the mechanical properties due to overconsumption of the double bonds [66]. Thus, optimizing the oxidation level and reaction conditions is essential for balancing enhanced functionality with preservation of the intrinsic elastomeric characteristics of PIP.

### 3.2. Hydroxyl Functionalization

Hydroxyl functionalization is an effective strategy for increasing the polarity and chemical reactivity of polyisoprene. The introduced hydroxyl groups can form hydrogen bonds and participate in subsequent reactions with isocyanates, carboxylic acids, epoxides, and other electrophilic reagents, providing versatile reactive sites for further molecular modification [60,61,71]. Consequently, hydroxyl-functionalized PIP generally exhibits enhanced intermolecular interactions, interfacial adhesion, and compatibility with polar polymers and inorganic fillers [30].

Hydroxyl groups can be introduced either through post-polymerization modification or by direct copolymerization with hydroxyl-containing monomers. In particular, stereoselective copolymerization provides a route to incorporate hydroxyl functionalities while retaining controlled PIP microstructures. Xu and co-workers prepared hydroxyl-functionalized PIP through copolymerization of isoprene with hydroxymyrcene using a cobalt catalytic system and hydroxyl-protecting alkylaluminum reagents. The resulting hydroxyl groups enhanced hydrogen-bonding interactions with polar fillers, leading to improved filler dispersion and mechanical performance [16].

Beyond direct reinforcement, hydroxyl groups serve as reactive molecular handles for secondary functionalization and network construction. Reactions involving pendant hydroxyl groups have been employed to introduce phosphate functionalities, biodegradable polyester segments, and polyurethane-type linkages, thereby enabling further regulation of interfacial interactions, mechanical properties, and polymer architecture [31,72]. Hydroxyl functionalization therefore plays a dual role in PIP molecular design: it strengthens intermolecular and interfacial interactions through hydrogen bonding while simultaneously providing reactive sites for subsequent chemical and network engineering.

However, increasing hydroxyl content does not necessarily lead to continuously improved performance. Excessive hydroxyl incorporation may strengthen intermolecular association to the extent that chain mobility and processability are compromised and may also promote undesirable side reactions during subsequent cross-linking [30]. Therefore, the hydroxyl content and its distribution along the PIP chains should be carefully controlled to balance interfacial reinforcement, chemical reactivity, and preservation of elastomeric chain dynamics.

### 3.3. Halogenation

Halogenation provides an effective post-polymerization route for increasing the polarity and chemical reactivity of polyisoprene. Halogen atoms can be introduced through electrophilic addition to carbon–carbon double bonds or allylic substitution using chlorine- or bromine-containing reagents, generating reactive C–Cl or C–Br functionalities [60,73]. These carbon–halogen bonds serve as versatile intermediates for subsequent nucleophilic substitution and coupling reactions, enabling the introduction of hydroxyl, amino, thiol, azide, phosphonate, and other functional groups [74,75].

More importantly, halogenated PIP can act as a reactive platform for subsequent macromolecular engineering. For example, brominated PIP has been employed as a macroinitiator for atom transfer radical polymerization (ATRP), while halogen-containing PIP can be converted into azide-functionalized derivatives for subsequent copper-catalyzed azide–alkyne cycloaddition (CuAAC) [76]. Halogenation therefore functions primarily as an intermediate activation strategy, converting the relatively inert hydrocarbon backbone of PIP into reactive sites for secondary functionalization, grafting, and click chemistry.

However, the extent of halogenation must be carefully controlled because excessive incorporation may reduce chain flexibility, promote thermal degradation or elimination reactions, and ultimately compromise elastomeric performance. In addition, the use of elemental halogens and certain halogenating reagents raises environmental and safety concerns. Thus, future development should emphasize selective and environmentally benign halogenation strategies that balance chemical reactivity with structural integrity and sustainability.

### 3.4. Click Chemistry

Click chemistry has emerged as an efficient and versatile post-polymerization modification strategy for the preparation of functional polyisoprene. Owing to its high efficiency, excellent chemoselectivity, mild reaction conditions, and broad functional-group tolerance, click chemistry enables rapid and quantitative functionalization of polyisoprene without significantly affecting its molecular architecture or elastomeric properties [55,75]. Compared with conventional modification methods, click reactions generally proceed with high yields under relatively simple reaction conditions, making them particularly attractive for the synthesis of complex polymer architectures and multifunctional elastomeric materials.

Among the various click reactions, thiol–ene click chemistry has attracted the greatest attention for polyisoprene functionalization because of the abundant carbon–carbon double bonds remaining in the polymer backbone. Under thermal or photo-induced radical conditions, thiol compounds can readily add across the carbon–carbon double bonds of polyisoprene with excellent efficiency and regioselectivity, enabling the introduction of a wide variety of functional groups, including hydroxyl, amino, carboxyl, fluorinated, and silane-containing moieties [77,78]. A recent study by Huang et al. further demonstrated the versatility of thiol–ene chemistry for constructing multifunctional PIP. L-Cysteine, containing thiol, carboxylic acid, and amine functionalities, was introduced into cis-1,4-PIP through a one-step radical-mediated thiol–ene reaction. The resulting functionalized PIP combined enhanced intermolecular interactions with self-healing and recyclable characteristics, illustrating how direct modification of backbone unsaturation can simultaneously introduce chemical functionality and dynamic material behavior [79]. Wang et al. extended thiol–ene functionalization to reactive polyisoprene network coatings prepared by electrodeposition. Polyisoprene containing a small fraction of electrochemically cross-linkable phenol groups was deposited as conformal ultrathin networks, while the remaining backbone unsaturation enabled subsequent thiol–ene modification. This strategy demonstrates that PIP functionalization can be integrated with surface and coating engineering, extending post-polymerization modification from bulk elastomers toward functional interfaces and complex substrates [80]. Furthermore, thiol–ene chemistry has been extensively employed to prepare cross-linked elastomeric networks, self-healing materials, and surface-functionalized polyisoprene owing to its nearly quantitative conversion and oxygen tolerance.

In addition to thiol–ene chemistry, copper(I)-catalyzed azide–alkyne cycloaddition (CuAAC) has become another representative click reaction for the construction of functional polyisoprene architectures. In this approach, azide- or alkyne-functionalized polyisoprene, typically obtained through prior halogenation, hydroxylation, or chain-end functionalization, can be efficiently coupled with complementary functional molecules to generate stable 1,2,3-triazole linkages [74,81]. Because CuAAC exhibits remarkable functional-group tolerance and proceeds under mild reaction conditions, it has been widely utilized for the synthesis of block copolymers, star polymers, graft copolymers, polymer brushes, and supramolecular assemblies with precisely controlled molecular structures. The resulting triazole units can further participate in hydrogen bonding or metal coordination, providing additional opportunities for constructing responsive polymer networks.

**Figure 3:**
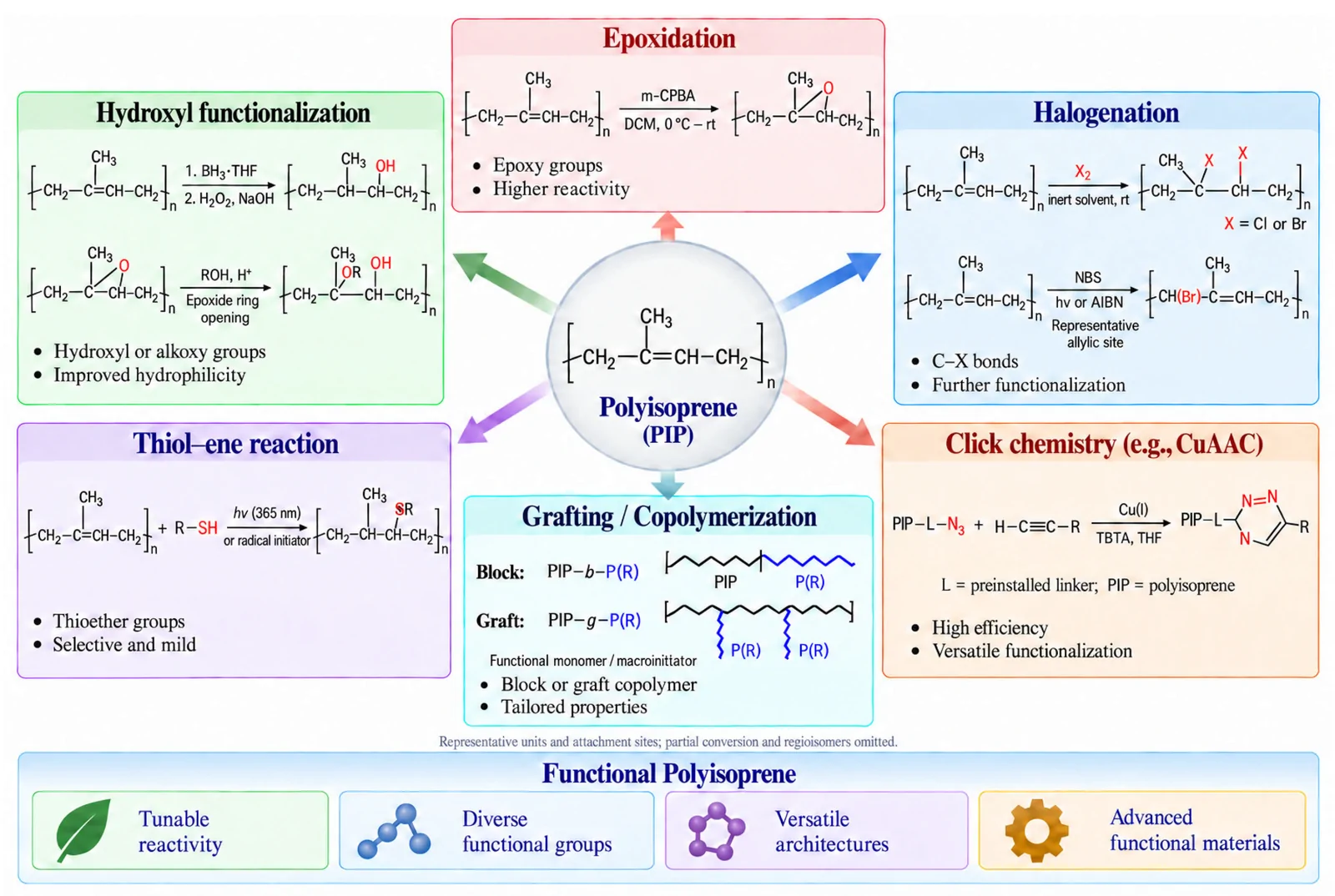
Major functionalization pathways of polyisoprene for molecular and network engineering.

Despite these significant advantages, several challenges remain. For CuAAC reactions, residual copper catalysts may be undesirable for biomedical applications and often require careful purification. Moreover, although thiol–ene reactions generally exhibit excellent selectivity, incomplete conversion or competing radical side reactions may occur under unsuitable reaction conditions [82]. Consequently, catalyst selection, reaction kinetics, and functional-group compatibility should be carefully optimized to maximize modification efficiency while preserving the molecular integrity of polyisoprene. Therefore, click chemistry serves as an efficient molecular bridge between the intrinsic unsaturation of PIP and the programmable installation of functional groups, polymer architectures, and dynamic network motifs. As presented in Figure 3, the intrinsic unsaturation of PIP provides a versatile platform for diverse functionalization pathways. Epoxidation, hydroxyl functionalization, halogenation, and click reactions introduce polar or reactive functionalities, while grafting and copolymerization further regulate macromolecular architecture. Together, these strategies provide the molecular basis for subsequent intermolecular interaction and network engineering.

### 3.5. Grafting and Macromolecular Architecture Engineering

Grafting provides a versatile strategy for introducing functional polymer segments into polyisoprene while preserving the elastomeric characteristics of the PIP backbone. Depending on how the graft chains are incorporated, grafting strategies can generally be classified as “grafting onto”, “grafting from”, and “grafting through”. These approaches offer different levels of control over grafting density and macromolecular architecture, enabling the construction of branched and brush-like PIP derivatives [83,84]. Functional polymer segments such as poly(acrylates), poly(methacrylates), poly(ethylene glycol), polystyrene, and biodegradable polyesters have been incorporated into PI-based architectures, providing improved interfacial compatibility and opportunities to introduce responsive, conductive, or bioactive functionalities.

Copolymerization provides a complementary means of regulating PIP architecture by incorporating additional polymer segments during synthesis. In particular, block copolymerization enables microphase-separated structures in which flexible PIP domains are combined with rigid or functional polymer blocks, thereby integrating elasticity with mechanical reinforcement and processability [85,86]. Copolymerization with polar functional monomers can additionally introduce hydroxyl, ester, ether, pyridine, amide, or phosphonate functionalities while maintaining controlled PIP microstructures, providing reactive sites for subsequent chemical modification and network construction [16,62,70,87].

From a molecular-design perspective, grafting and copolymerization extend PIP functionalization beyond the introduction of individual functional groups by enabling control over macromolecular architecture and spatial distribution of functionality. Graft density, block composition, sequence distribution, and phase morphology can therefore regulate interfacial interactions, stress transfer, chain mobility, and subsequent cross-linking behavior. However, increasing architectural complexity may also introduce steric constraints, phase incompatibility, or processing challenges. Thus, rational control of polymer architecture is essential for translating molecular functionalization into desirable macroscopic properties.

This architectural control also provides an important bridge to network engineering, because functional segments and reactive sites introduced through grafting or copolymerization can subsequently participate in supramolecular interactions and dynamic cross-linking. Consequently, the design of polymer architecture and the construction of dynamic networks should be considered complementary levels of functional PIP engineering. As shown in Table 2, the functionalization strategies discussed above provide complementary approaches for tailoring the chemical structure and macromolecular architecture of PIP. Epoxidation, hydroxyl functionalization, and halogenation primarily introduce reactive sites for subsequent modification, whereas click chemistry enables efficient and selective installation of diverse functionalities. Grafting and copolymerization further extend molecular functionalization toward architectural control. The selection of an appropriate strategy therefore depends on the desired balance among functionalization efficiency, structural integrity, chemical versatility, and subsequent network-forming capability.

### 3.6. Dynamic Crosslinking and Network Formation

Conventional sulfur vulcanization has long been the dominant cross-linking strategy for polyisoprene owing to its ability to produce elastomers with outstanding elasticity, tensile strength, and fatigue resistance. However, the permanent covalent cross-links formed during sulfur vulcanization are irreversible, making the resulting materials difficult to reprocess, repair, or recycle. These intrinsic limitations have become increasingly incompatible with the growing demand for sustainable and intelligent polymeric materials. Consequently, the development of dynamic cross-linking strategies has emerged as a major research direction for transforming functionalized polyisoprene into next-generation smart elastomers.

Unlike conventional thermosets, dynamic polymer networks contain reversible chemical or physical cross-links that can dissociate and reform in response to external stimuli, including heat, light, mechanical force, moisture, or changes in pH. Such reversible bond exchange enables network rearrangement without completely destroying the cross-linked structure, thereby imparting unique properties such as self-healing, recyclability, reprocessability, shape-memory behavior, and adaptive mechanical responses [89,90].

Dynamic cross-linking strategies can generally be divided into dynamic covalent networks and supramolecular networks. Dynamic covalent networks rely on reversible covalent bond exchange reactions, including disulfide exchange, Diels–Alder cycloaddition, imine formation, boronic ester exchange, transesterification, and acylhydrazone chemistry [91,92,93]. Among these, disulfide bonds are particularly attractive because they undergo rapid exchange under thermal or redox conditions, enabling efficient self-healing and reprocessing while maintaining satisfactory mechanical strength. Similarly, thermally reversible Diels–Alder reactions provide a convenient route for constructing polymer networks that can repeatedly undergo cross-linking and decross-linking upon heating and cooling, making them highly suitable for recyclable elastomeric materials.

In recent years, vitrimer technology has attracted considerable attention as an emerging class of dynamic polymer networks. Unlike dissociative dynamic networks, vitrimers operate through associative bond-exchange mechanisms, allowing network topology to rearrange while maintaining an essentially constant cross-link density [94,95]. As a result, vitrimer-like polyisoprene networks combine the dimensional stability and mechanical robustness of conventional thermosets with the reprocessability of thermoplastics. These materials can be reshaped, repaired, and recycled without significant loss of mechanical performance, providing promising solutions for sustainable rubber manufacturing.

Besides dynamic covalent chemistry, supramolecular interactions offer another effective strategy for constructing adaptive polyisoprene networks. Reversible hydrogen bonding, ionic interactions, metal–ligand coordination, host–guest recognition, and π–π stacking interactions can serve as dynamic physical cross-links that reversibly dissociate and reform under external stimuli [96,97,98]. Compared with covalent dynamic networks, supramolecular elastomers generally exhibit faster response rates and milder healing conditions, although their mechanical strength is often lower. Combining multiple supramolecular interactions or integrating supramolecular interactions with dynamic covalent chemistry has therefore become an effective strategy for simultaneously improving toughness, self-healing efficiency, and environmental adaptability.

The availability of functional groups introduced through epoxidation, hydroxylation, halogenation, click chemistry, and grafting provides abundant reactive sites for constructing these dynamic polymer networks. Hydroxyl groups readily participate in polyurethane formation and transesterification reactions; epoxy groups can be incorporated into vitrimer networks; and azide–alkyne click chemistry enables precise installation of dynamic cross-linking motifs. Consequently, chemical functionalization and dynamic network construction should be regarded as complementary strategies rather than independent modification approaches. Their integration enables precise regulation of cross-link density, bond-exchange kinetics, and network topology, ultimately determining the balance between elasticity, mechanical strength, and dynamic functionality.

**Figure 4:**
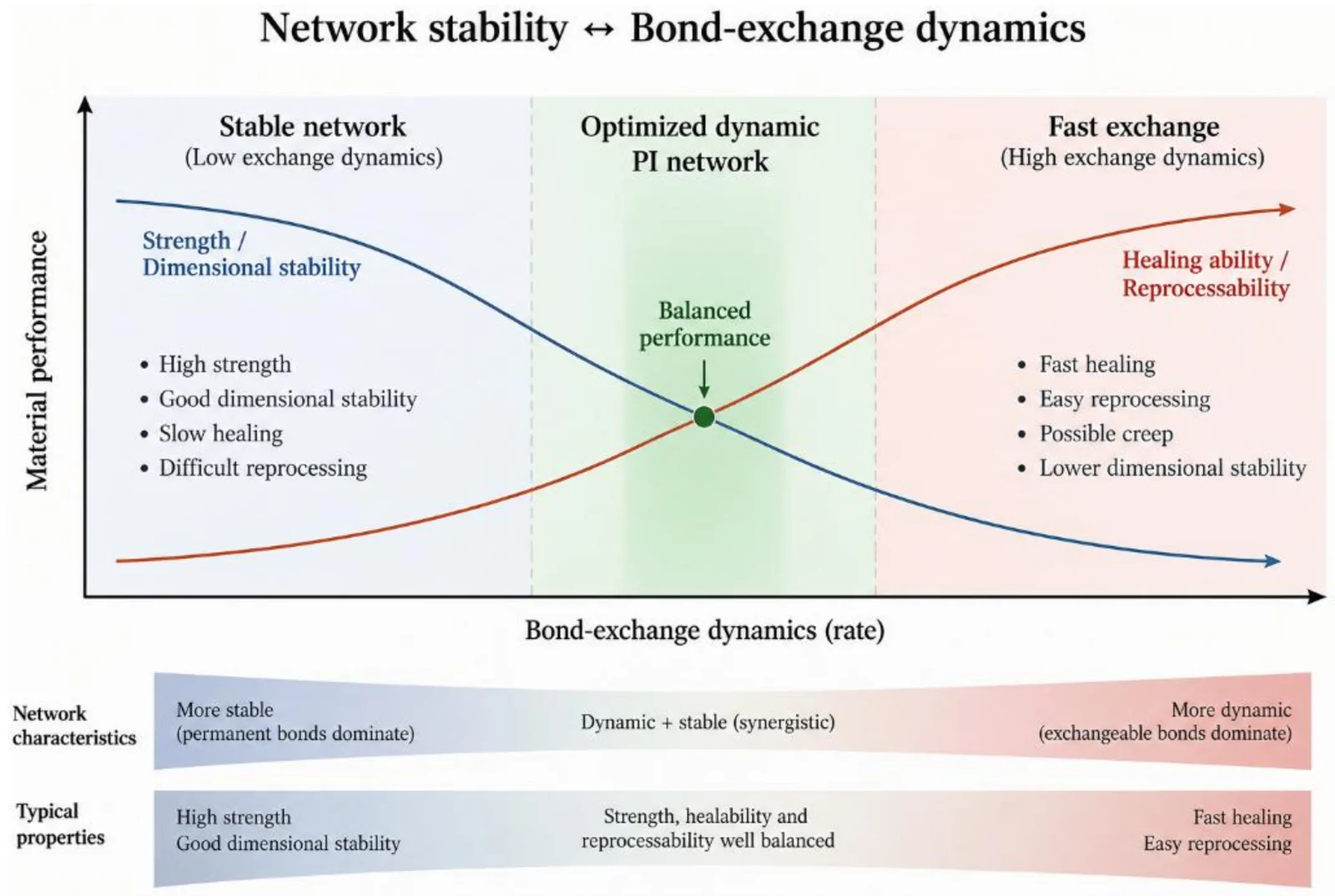
Trade-off between network stability and bond-exchange dynamics in functional polyisoprene-based dynamic networks.

As illustrated in Figure 4, the performance of dynamic polyisoprene networks is governed by a fundamental trade-off between network stability and bond-exchange dynamics. Networks dominated by stable cross-links generally exhibit high mechanical strength and good dimensional stability, but restricted network rearrangement results in slow healing and limited reprocessability. In contrast, increasing the rate of dynamic bond exchange facilitates network topology rearrangement, thereby accelerating self-healing and enabling efficient reprocessing. Excessively rapid bond exchange, however, may promote stress relaxation and creep, leading to reduced dimensional stability and long-term mechanical robustness. Therefore, the design of high-performance dynamic PIP elastomers should not simply maximize the density or exchange rate of dynamic bonds. Instead, an appropriate balance between stable network constraints and reversible bond exchange is required to simultaneously achieve mechanical robustness, efficient healing, reprocessability, and dimensional stability.

## 4. Functional Polyisoprene-Based High-Performance and Smart Elastomers

The incorporation of functional groups into polyisoprene (PIP) chains has enabled the development of advanced elastomeric materials with enhanced performance and novel functionalities. In this review, “functional PIP” is used as a broad term for PIP materials endowed with additional properties through chemical or structural modification, whereas “smart elastomers” specifically refer to functional PIP systems capable of advanced responses or adaptation. Within this context, “dynamic” describes reversible molecular interactions or network rearrangements, while “stimuli-responsive” refers to measurable changes in material properties or shape-induced changes by external stimuli. The term “adaptive” is used more broadly for materials capable of adjusting their structure or properties in response to environmental or mechanical changes. These concepts are therefore related but are not considered interchangeable. By tailoring the chemical structure through various modification strategies, it is possible to precisely regulate intermolecular interactions, crosslinking behavior, and network architecture. As a result, functionalized polyisoprene has emerged as a versatile platform for the design of high-performance and smart elastomers. This section highlights recent progress in functionalized PIP-based elastomers, with emphasis on the relationship between chemical modification and material properties.

### 4.1. Mechanically Reinforced Elastomers

One of the primary objectives of polyisoprene functionalization is to develop elastomers with simultaneously enhanced mechanical strength, toughness, and durability while retaining the excellent elasticity characteristic of conventional rubber. Although cis-1,4-polyisoprene exhibits remarkable resilience and flexibility, its intrinsically nonpolar backbone and relatively weak intermolecular interactions often result in limited filler compatibility, poor interfacial adhesion, and insufficient mechanical reinforcement. Functionalization therefore provides an effective strategy to strengthen intermolecular interactions and optimize network structures, leading to significant improvements in the mechanical performance of polyisoprene-based elastomers [99].

The enhancement of mechanical properties generally originates from three complementary mechanisms: (i) increasing intermolecular interactions through the introduction of polar functional groups; (ii) improving filler dispersion and filler–rubber interfacial adhesion; and (iii) constructing optimized cross-linked network architectures. Polar groups such as hydroxyl, epoxy, carboxyl, phosphonate, and pyridine moieties can introduce hydrogen bonding, dipole–dipole interactions, ionic interactions, or coordination interactions between polymer chains, thereby increasing cohesive energy density and facilitating more efficient stress transfer under deformation [16,30,31].

Among the various modification strategies, hydroxyl-functionalized polyisoprene has demonstrated excellent reinforcement capability. Xu and co-workers reported the copolymerization of isoprene with hydroxyl-containing myrcene derivatives using a stereoselective cobalt catalytic system. The incorporated hydroxyl groups significantly enhanced hydrogen-bonding interactions between the rubber matrix and polar fillers, resulting in improved filler dispersion and remarkable increases in tensile strength and modulus without severely compromising elasticity. Similarly, epoxidized polyisoprene exhibits stronger interactions with silica, carbon black, and other reinforcing fillers owing to its increased polarity, leading to improved interfacial adhesion, reduced filler aggregation, and enhanced mechanical performance [100,101]. Zhang et al. employed phosphatidylcholine to regulate lignin polarity and promote the formation of cell-like self-assembled structures within hydroxylated PIP. The resulting interfacial layer improved compatibility and stress transfer between lignin and the rubber matrix, enabling a tensile strength of approximately 26 MPa without permanent chemical cross-linking [102]. This study highlights how functional-group-mediated interfacial assembly can provide an additional route for simultaneously reinforcing and toughening PIP elastomers.

Grafting and block copolymerization further provide effective approaches for mechanical reinforcement by regulating phase morphology and molecular architecture. The introduction of rigid polymer segments or hard domains through graft copolymerization promotes efficient stress dissipation while maintaining the flexibility of the polyisoprene matrix. In block copolymer systems, microphase-separated morphologies consisting of soft polyisoprene domains and rigid thermoplastic segments simultaneously contribute to high tensile strength, toughness, and excellent processability, making these materials attractive candidates for thermoplastic elastomers [72,85,86].

Recent studies have demonstrated that the integration of dynamic interactions into reinforced elastomers can further improve toughness without sacrificing recoverability. Dynamic hydrogen bonding, metal–ligand coordination, ionic associations, and reversible covalent bonds function as sacrificial interactions that dissipate mechanical energy during deformation and subsequently reform after unloading, thereby enhancing fracture resistance and fatigue durability [9,103,104]. Such synergistic reinforcement mechanisms effectively overcome the traditional trade-off between strength and elasticity that commonly exists in conventional chemically cross-linked rubbers.

The structure–property relationship of reinforced polyisoprene is governed by multiple molecular parameters, including functional group type, functionalization degree, molecular weight, cross-link density, filler dispersion, and network topology. Increasing the density of polar groups generally strengthens intermolecular interactions and improves filler compatibility; however, excessive functionalization may reduce chain mobility, impair strain-induced crystallization, and consequently decrease elasticity. Likewise, higher cross-link densities increase modulus and tensile strength but may lead to reduced elongation at break and toughness. Therefore, rational molecular engineering should balance these competing factors to achieve optimal comprehensive mechanical performance.

### 4.2. Self-Healing Elastomers

Self-healing elastomers are attractive because they can repair mechanical damage and thereby extend material lifetime and reliability. Conventional sulfur-vulcanized PIP contains irreversible covalent cross-links that restrict chain rearrangement and crack repair. In contrast, functionalized PIP can incorporate reversible dynamic interactions, providing a route to elastomers that combine elasticity with intrinsic self-healing capability [105,106].

Self-healing in functional PIP primarily relies on the reversible dissociation and reformation of dynamic cross-links after damage. Dynamic covalent bonds, such as disulfide and Diels–Alder linkages, can reconstruct damaged networks through bond exchange or reversible reactions, whereas supramolecular interactions, including hydrogen bonding, ionic association, and metal–ligand coordination, enable rapid network reformation through reversible noncovalent interactions [106,107,108]. In general, dynamic covalent networks provide relatively robust network structures, while supramolecular interactions often enable faster healing under milder conditions. Hybrid networks combining multiple reversible interactions therefore provide a promising strategy for balancing healing kinetics with mechanical performance. More recently, Laysandra et al. developed radically modified cis-1,4-PIP by incorporating N-vinyl carbazole and 2-[[(butylamino)carbonyl]oxy]ethyl acrylate, thereby combining π–π stacking and dynamic hydrogen-bonding interactions. The optimized material exhibited a tensile stress of 2.91 MPa, a maximum strain of 1087%, and a toughness of 25.27 MJ m^−3^, while maintaining 96% of its initial dissipated energy after 25 cyclic tests. Importantly, autonomous self-healing efficiencies above 90% were achieved, together with solution-based recyclability [109]. Li et al. demonstrated that regulating the composition and content of sulfide bonds provides another effective route for balancing mechanical robustness, self-healing, and recyclability in PIP. By introducing vulcanization modifiers, tensile strengths of approximately 30 MPa were achieved at relatively low modifier contents, whereas increasing the fraction of exchangeable sulfide bonds enabled a maximum self-healing efficiency of 89.8%. After remolding, the tensile-strength recovery reached 104%, with an elongation-at-break recovery of 82.6% [110].

Importantly, self-healing performance is governed not simply by the concentration of reversible bonds but by the interplay among chain mobility, interaction strength, and bond-exchange kinetics. Sufficient chain mobility is required for damaged interfaces to reconnect, whereas reversible interactions must be strong enough to restore network integrity after healing. Functionalization therefore provides a means of regulating both the density of dynamic cross-linking sites and the local molecular environment, enabling self-healing behavior to be optimized through molecular and network design [103].

However, improving healing efficiency often involves a trade-off with mechanical robustness and dimensional stability. Increasing the density or exchange rate of reversible bonds can accelerate network reconstruction but may reduce stiffness and creep resistance, whereas highly cross-linked networks restrict chain mobility and consequently slow healing. Therefore, future development should focus on achieving rapid healing under mild or ambient conditions while maintaining mechanical strength, fatigue resistance, dimensional stability, and healing performance over repeated damage–repair cycles [7,105].

### 4.3. Reprocessable and Recyclable Elastomers

The development of reprocessable and recyclable elastomers is increasingly important for reducing material waste and improving the sustainability of rubber materials. Conventional sulfur-vulcanized PIP contains permanent covalent networks that prevent remolding after cross-linking. In contrast, functionalized PIP can incorporate dynamic covalent bonds or reversible supramolecular interactions and allow network rearrangement while maintaining sufficient cross-link density for mechanical integrity [94,111,112,113].

Dynamic PIP networks provide a particularly promising route to material reprocessing. Vitrimer-like networks can undergo topology rearrangement through associative bond exchange, enabling reshaping and reprocessing by thermal and pressure-assisted treatments while retaining network integrity [114]. Reversible supramolecular interactions can similarly facilitate network reconstruction under relatively mild conditions, although their lower interaction strength may limit mechanical stability. These approaches therefore provide complementary strategies for introducing reprocessability into cross-linked PIP.

Reprocessing performance is governed by the balance among bond-exchange kinetics, chain mobility, and network stability. Faster dynamic exchange facilitates stress relaxation and reshaping, whereas excessive exchange may compromise creep resistance and dimensional stability. Conversely, highly stable networks provide mechanical robustness but require more demanding reprocessing conditions. Rational regulation of cross-link density and exchange kinetics is therefore essential for balancing mechanical performance, reprocessability, and long-term durability.

Despite recent progress, several challenges remain, including maintaining mechanical properties over repeated reprocessing cycles, accelerating network rearrangement under mild conditions, reducing catalyst requirements, and developing scalable processing strategies [111]. Future efforts should also integrate reprocessable network chemistry with renewable feedstocks and other adaptive functionalities to advance more sustainable PIP-based elastomers.

### 4.4. Shape-Memory and Stimuli-Responsive Elastomers

Shape-memory elastomers (SMEs) have attracted increasing attention because they can recover their original shape after deformation upon exposure to external stimuli such as heat, light, electricity, or magnetic fields. Compared with conventional shape-memory polymers, elastomeric systems exhibit larger reversible deformation, lower elastic modulus, and better fatigue resistance, making them highly attractive for applications in soft robotics, biomedical devices, flexible electronics, and aerospace engineering [115,116,117].

Functionalized polyisoprene provides an ideal platform for constructing shape-memory elastomers owing to its excellent elasticity and highly tunable molecular architecture. The shape-memory behavior of polyisoprene-based elastomers generally relies on the coexistence of permanent network structures that determine the original shape and reversible switching domains that temporarily fix the deformed configuration. Functional groups introduced through copolymerization or post-polymerization modification facilitate the construction of such dual-network systems by incorporating crystalline domains, hydrogen-bonding interactions, or thermally reversible dynamic covalent bonds [118].

Recent studies have demonstrated that Diels–Alder chemistry, hydrogen bonding, and vitrimer-like dynamic networks can effectively endow polyisoprene with excellent shape-memory performance while maintaining high elasticity and mechanical strength [108,113]. Block copolymerization also provides an efficient strategy for generating microphase-separated morphologies, in which rigid domains serve as permanent cross-linking points whereas polyisoprene segments function as reversible elastic matrices. Consequently, shape fixation and shape recovery can be achieved with high efficiency over multiple deformation cycles.

Despite these advances, achieving rapid shape recovery together with excellent mechanical robustness and long-term cycling stability remains a major challenge. Future efforts are expected to focus on integrating multiple dynamic interactions and designing multi-responsive polyisoprene networks capable of responding to thermal, optical, electrical, and magnetic stimuli simultaneously. As summarized in Table 3, different functional PIP elastomers rely on distinct molecular and network-design strategies to achieve mechanical reinforcement, self-healing, reprocessability, and stimuli responsiveness. Despite their different functions, these systems share a common design principle: macroscopic performance is governed by the balance among intermolecular interactions, chain mobility, network stability, and dynamic bond exchange.

## 5. Structure–Property Relationships and Molecular Design Principles

Following the hierarchical framework introduced in Figure 1, this section further integrates the relationships among molecular structure, intermolecular interactions, network architecture, and macroscopic properties to identify general design principles for functional PIP elastomers. The outstanding performance of functionalized polyisoprene originates from the intimate relationship between molecular structure, network architecture, and macroscopic material properties. Unlike conventional elastomers, whose properties are mainly determined by cross-link density, functionalized polyisoprene provides multiple molecular design parameters, including functional group type, functionalization degree, stereoregularity, molecular architecture, filler interface, and dynamic network topology [12,119,120]. Recent molecular-level studies further indicate that network properties are governed not only by cross-link density but also by the detailed architecture of the cross-links. Ghanta et al. investigated cross-linked PIP networks with defined sulfide-linker architectures using all-atom molecular models, demonstrating the importance of linker structure and connectivity in regulating network characteristics, thermodynamics, and microscopic dynamics [121]. These results reinforce the need to consider cross-link chemistry and topology, rather than cross-link density alone, when establishing quantitative structure–property relationships in PIP elastomers. Generally, increasing the content of polar functional groups, enhances intermolecular interactions and filler compatibility, leading to improved mechanical strength and interfacial adhesion. However, excessive functionalization may reduce chain flexibility and strain-induced crystallization, thereby decreasing elasticity. Likewise, higher cross-link densities increase stiffness and tensile strength but often sacrifice elongation at break and chain mobility.

The incorporation of dynamic covalent bonds and supramolecular interactions introduces an additional design dimension by enabling simultaneous regulation of mechanical performance, self-healing capability, recyclability, and stimuli responsiveness. Consequently, the overall performance of functionalized polyisoprene results from balancing chain mobility, intermolecular interactions, network stability, and bond exchange kinetics rather than optimizing any single structural parameter.

Future molecular design should therefore emphasize integrated optimization rather than individual functionalization strategies. Combining precision polymerization, controlled functionalization, hierarchical network construction, and multiscale structural regulation is expected to provide new opportunities for developing high-performance smart elastomers capable of simultaneously exhibiting excellent mechanical properties, environmental adaptability, sustainability, and multifunctionality.

**Figure 5:**
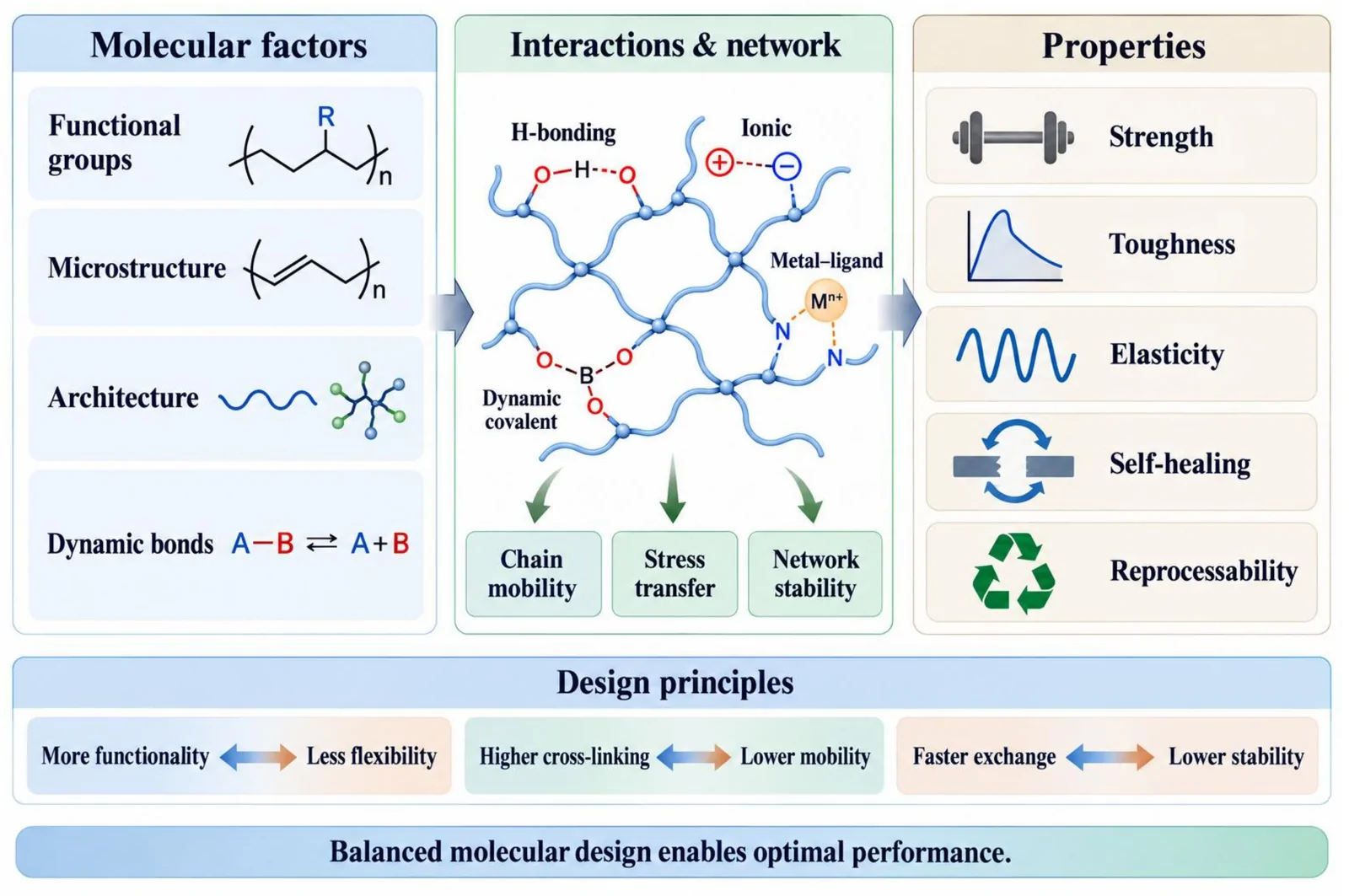
Molecular design map illustrating the structure–property relationships of functional polyisoprene elastomers.

As shown in Figure 5, the macroscopic performance of functional PIP elastomers arises from the coupled effects of molecular structure, intermolecular interactions, and network architecture. Functional groups, microstructure, macromolecular architecture, and dynamic bonds collectively regulate chain mobility, stress transfer, and network stability, thereby determining mechanical strength, toughness, elasticity, self-healing, and reprocessability. Rational molecular design therefore requires balancing these interconnected parameters rather than maximizing any single structural feature.

## 6. Emerging Applications

Although this review primarily focuses on functional synthetic polyisoprene, direct demonstrations of some emerging applications remain relatively limited. Therefore, selected studies on natural rubber (NR), epoxidized natural rubber (ENR), and closely related polyisoprene-based systems are also discussed in this section when they provide transferable design concepts for functional PIP. These examples are used to illustrate potential structure–function relationships and application strategies rather than as direct evidence for synthetic PIP. Accordingly, most of the emerging applications discussed below should currently be regarded as proof-of-concept or application-potential demonstrations rather than technologies ready for large-scale implementation. Application readiness should be evaluated using application-specific metrics, including sensitivity, gauge factor, response and recovery times, cycling stability, hysteresis, conductivity retention after damage/healing, and long-term environmental stability for flexible sensing systems, as well as biosafety, sterilization compatibility, and manufacturing scalability for biomedical applications.

### 6.1. Flexible Electronics and Sensors

The rapid development of wearable electronics, human–machine interfaces, and soft robotics has stimulated growing demand for elastomeric materials possessing excellent flexibility, mechanical durability, and electrical functionality. Conventional polyisoprene exhibits outstanding elasticity but lacks electrical conductivity and sensing capability. Functionalization provides an effective strategy for integrating conductive fillers, responsive functional groups, and dynamic polymer networks into polyisoprene, thereby enabling its application in flexible electronic devices [122,123].

Functionalized polyisoprene has been widely employed as an elastic matrix for conductive composites containing carbon nanotubes, graphene, MXene, carbon black, or metallic nanowires. The incorporation of polar functional groups significantly improves interfacial compatibility between the polymer matrix and conductive fillers, resulting in more homogeneous filler dispersion and more stable conductive networks. Consequently, the resulting composites exhibit excellent electrical conductivity together with high stretchability and mechanical durability [124,125].

Recent studies have demonstrated that combining dynamic polymer networks with conductive fillers enables the fabrication of self-healing strain sensors capable of restoring both mechanical integrity and electrical conductivity after damage. These multifunctional elastomers show excellent sensitivity, rapid response, and stable signal output under repeated deformation, making them promising candidates for wearable health monitoring, electronic skin, and intelligent sensing devices [126]. For practical implementation, future studies should systematically report standardized sensing metrics, including sensitivity or gauge factor, response and recovery times, hysteresis, cycling durability, conductivity retention after damage and healing, and stability under variations in temperature and humidity. At present, insufficient long-term and standardized performance evaluation remains a major barrier to translating functional PIP-based sensing materials from laboratory demonstrations to practical devices. A recent review by Li et al. highlighted conjugated polyisoprene as an emerging platform for inherently stretchable conducting materials, with potential applications in stretchable conductors, strain sensors, flexible energy-storage devices, and electrocatalysis. Strategies including catalytic isomerization, oxidative dehydrogenation, and halogen-doping-induced conjugation have been explored to introduce electronic functionality into PIP-based rubbers. Nevertheless, limited charge mobility, dopant instability, and scalability remain important barriers to practical implementation [127].

### 6.2. Biomedical Applications

The excellent elasticity and tunable chemical functionality of polyisoprene provide a useful platform for developing biomedical elastomeric materials. Nevertheless, the inherent hydrophobicity and limited bioactivity of conventional polyisoprene restrict its direct application in biological environments. Functionalization therefore plays a crucial role in improving its biological performance by introducing hydrophilic groups, biodegradable segments, or bioactive molecules [128,129,130].

Hydroxyl, carboxyl, amino, and polyethylene glycol (PEG) functionalities have been extensively incorporated into polyisoprene to improve hydrophilicity, protein resistance, and cellular compatibility. In addition, bioactive peptides, polysaccharides, and antimicrobial molecules can be covalently immobilized onto functionalized polyisoprene through click chemistry or other coupling reactions, enabling precise regulation of cell adhesion and biological interactions [131].

Related natural-rubber-derived systems have further demonstrated the potential of elastomeric networks for biomedical applications, including tissue engineering and soft-tissue-related materials [132]. Although these studies are not based on synthetic PIP, they provide useful design concepts for introducing biological functionality and adaptability into future functional PIP systems. Therefore, biomedical applications of functional synthetic PIP should currently be considered primarily at the exploratory stage, and substantial validation of long-term biosafety, sterilization tolerance, degradation behavior, and manufacturing reproducibility is still required before clinical translation.

## 7. Challenges and Future Perspectives

The development of precision polymerization, post-polymerization modification, and dynamic network engineering has greatly expanded the molecular design of functionalized polyisoprene (PIP). Compared with conventional synthetic PIP, functionalized PIP exhibits improved mechanical properties and multifunctional characteristics, including self-healing, reprocessability, stimuli responsiveness, and shape-memory behavior. These advances have established PIP as a promising platform for next-generation smart elastomers. Nevertheless, several challenges remain for its further development and practical application.

The literature reviewed above reveals substantial progress in the precision synthesis, functionalization, and dynamic network engineering of polyisoprene, while also identifying several unresolved challenges that currently limit the transition from molecular design to practical applications. From the perspective of the hierarchical framework developed in this review, these challenges can be broadly associated with three interconnected levels: achieving precise molecular functionalization without compromising the intrinsic elasticity and stereoregularity of PIP; establishing quantitative structure–property relationships between molecular/network parameters and macroscopic performance; and translating multifunctional laboratory-scale materials into scalable and durable elastomeric systems. Addressing these issues will be central to the future development of functional PIP.

The first challenge is the precise regulation of molecular structures while maintaining the intrinsic elasticity of PIP. Although various functionalization strategies have been developed, the introduction of functional groups may affect catalyst activity, stereoregularity, molecular weight control, and chain mobility. Therefore, future efforts should focus on developing highly efficient catalyst systems and selective functionalization approaches that enable precise molecular modification without sacrificing the fundamental mechanical properties of PIP.

Another challenge is the insufficient understanding of structure–property relationships. Although functional groups, cross-link density, and network architectures are known to influence the performance of PIP elastomers, the underlying molecular mechanisms remain unclear. Integrating advanced characterization techniques, molecular simulations, and theoretical modeling will be essential for establishing predictive design principles and achieving rational control over mechanical strength, toughness, self-healing, and recyclability.

The development of multifunctional smart elastomers represents another important direction. Current functionalized PIP systems are often designed to improve individual properties, whereas future applications require the integration of multiple functions, such as self-healing, recyclability, conductivity, sensing capability, and environmental adaptability. Achieving this goal will require the synergistic design of molecular structures, dynamic networks, and hierarchical architectures.

A central contribution of this review is therefore the integration of these previously dispersed research directions into a hierarchical molecular-design framework linking precision polymerization and microstructure control with functionalization, intermolecular interactions, network architecture, and ultimately macroscopic performance. Rather than considering polymer synthesis, chemical modification, and smart functionality as independent topics, this framework emphasizes their sequential and interdependent relationships. Such an integrated perspective provides a basis for identifying molecular-design trade-offs and for guiding the development of PIP elastomers with balanced mechanical performance, dynamic functionality, processability, and application potential.

Sustainability is also a critical consideration for the future development of functionalized PIP. Although dynamic networks have enabled recyclable elastomers, most PIP materials still rely on petroleum-derived isoprene and conventional catalytic systems. Future research should explore renewable feedstocks, environmentally benign catalysts, solvent-free modification strategies, and energy-efficient recycling processes. The integration of sustainable synthesis with precision polymerization and dynamic network engineering will provide new opportunities for environmentally responsible high-performance elastomers. Future advances in functionalized PIP will depend on the integration of molecular design, precise synthesis, dynamic network construction, and sustainable manufacturing, enabling the transition of PIP-based elastomers toward multifunctional, intelligent, and sustainable polymer materials.

Based on these challenges, future research is expected to evolve from single-function optimization toward integrated molecular and network design. Particular priorities include the development of catalyst systems that combine stereochemical precision with functional-group tolerance, quantitative and predictive structure–property models, dynamic networks capable of balancing mechanical robustness with rapid and repeatable adaptation, multifunctional systems integrating self-healing, sensing, conductivity, and recyclability, and scalable manufacturing strategies based on renewable feedstocks and energy-efficient processing. Progress along these directions will be essential for translating functional PIP from molecularly engineered laboratory materials toward practical, multifunctional, and sustainable elastomeric systems.

## Figures and Tables

**Table 1:** Comparison of major precision polymerization strategies for functional polyisoprene.

Polymerization Strategy	Stereochemical Control	Molecular-Weight Control	Functional-Group Tolerance	Scalability	Main Advantage	Limitations	Refs.
Rare-earth coordination	Excellent; cis-1,4 content typically > 97%	Good	Functional comonomer	High	High stereoselectivity	Catalyst poisoning by polar groups	[6,14,15,21,22,23,24,25,26,27,28,29,30,31]
CCTP	Excellent	Excellent	Chain-end	High	Efficient chain transfer; multiple chains per catalytic center	CTA/catalyst dependence	[32,33,35,36]
Living anionic	Moderate–good	Excellent	Chain-end	Low–Moderate	Precise molecular weight, narrow dispersity, high chain-end fidelity	Stringent conditions	[38,39,40,41,42,43,48,49]
ATRP	Limited	Good	Chain-end/block	Moderate	Functional-group tolerance	Limited PIP stereocontrol	[44,45]
RAFT	Limited	Good	Block/end groups	Moderate	Mild and versatile; suitable for block/end-group design	Limited stereocontrol	[44,46,47,50,51]

**Table 2:** Comparison of major functionalization strategies for polyisoprene.

Strategy	Reactive Site/Functionality	Key Effects	Advantages	Limitations	Refs.
Epoxidation	Backbone C=C → epoxide	Enhanced polarity, filler compatibility, and reactivity	Mature route; epoxide enables ring-opening and further functionalization	Over-epoxidation may cause chain scission and undesired cross-linking	[65,66,67,68,69,70]
Hydroxyl functionalization	Post-modification or copolymerization → –OH	Enhanced H-bonding, polarity, and interfacial adhesion	Reactive handle for esterification, urethane formation, and network construction	Excessive –OH may restrict chain mobility and processability	[16,30,31,71,72]
Halogenation	C=C/allylic sites → C–Cl or C–Br	Activates PIP for secondary modification	Enables substitution, azidation, ATRP, and CuAAC	Possible degradation; environmental and safety concerns	[73,74,75,76]
Thiol–ene click	C=C + R–SH → thioether functionality	Tunable polarity and interfacial properties	Efficient, mild, and directly uses PIP unsaturation	Possible incomplete conversion and radical side reactions	[55,75,77,78]
CuAAC click	Azide/alkyne-functionalized PIP → triazole	Precise coupling and architecture construction	High chemoselectivity and functional-group tolerance	Requires prefunctionalization; residual Cu may require removal	[62,74,81,82,88]
Grafting	Backbone/chain-end sites → polymer side chains	Controls interface, phase morphology, and functionality distribution	Enables graft, brush, and hierarchical architectures	Steric hindrance, phase incompatibility, and synthetic complexity	[83,84]
Functional copolymerization	Direct functional-comonomer incorporation	Tunable polarity and functionality distribution	Integrates synthesis and functionalization; stereocontrol can be retained	Polar monomers may deactivate catalysts	[16,62,85,86,87]

**Table 3:** Comparison of representative functional polyisoprene-based high-performance and smart elastomers.

Function	Network Strategy	Mechanism	Key Performance	Main Trade-Off	Refs.
Mechanically reinforced	Polar groups, filler interfaces, graft/block architectures, and sacrificial interactions	Enhanced interfacial interactions, stress transfer, and energy dissipation	Improved strength, toughness, filler dispersion, and fatigue resistance; OH-functionalized PI/SiO_2_: 9.6 MPa tensile strength and 885% elongation	Excessive functionalization/cross-linking may restrict chain mobility and strain-induced crystallization (SIC)	[16,30,31,85,99,100,101,102,103,104]
Self-healing	Dynamic covalent and supramolecular networks	Reversible bond exchange and network reconstruction	Damage repair and mechanical recovery; H-bond/Zn–triazole PI: 21 MPa tensile strength, recovering to 15.5 MPa after healing	Faster exchange favors healing but may reduce stiffness, creep resistance, and dimensional stability	[103,105,106,107,108,109,110]
Reprocessable and recyclable	Vitrimer, reversible Diels–Alder, and supramolecular networks	Bond exchange and topology rearrangement	Reshaping and reprocessing; Diels–Alder-cross-linked cis-1,4-PI retained performance over five reshaping cycles	Rapid exchange may increase creep; stable networks require harsher reprocessing conditions	[94,111,112,113,114]
Shape-memory and stimuli-responsive	Permanent networks with reversible/crystalline switching domains	Shape fixation and stimulus-triggered recovery	Reversible shape programming; ENR vitrimer showed >95% shape fixation and recovery	Trade-off among recovery rate, mechanical robustness, and cycling stability	[115,116,117,118]

## Data Availability

The original contributions presented in this study are included in the article. Further inquiries can be directed to the corresponding author.

## Abbreviations

The following abbreviations are used in this manuscript:

PIP	Polyisoprene
NR	Natural rubber
CCTP	Coordinative chain-transfer polymerization
ATRP	Atom transfer radical polymerization
RAFT	Reversible addition–fragmentation chain transfer
CuAAC	Copper(I)-catalyzed azide–alkyne cycloaddition
ENR	Epoxidized natural rubber
SIC	Strain-induced crystallization
